# An Intact Dopamine Sensitivity in the Brain: A Necessity to Recover Hyperprolactinemia and Galactorrhea in a Female Hemodialysis Patient?

**DOI:** 10.1155/2017/3729629

**Published:** 2017-07-16

**Authors:** Eva Philipse, Ester Philipse, Theodorus Twickler, Amaryllis Van Craenenbroeck, Marie Madeleine Couttenye, Luc Van Gaal

**Affiliations:** ^1^Department of Endocrinology, University Hospital of Antwerp, Edegem, Belgium; ^2^Department of Nephrology, University Hospital of Antwerp, Edegem, Belgium

## Abstract

A female hemodialysis patient with galactorrhea due to hyperprolactinemia was treated with different dialysis modalities to assess the effect on prolactin levels. A single session of both high-flux hemodialysis and hemodiafiltration resulted in decreased prolactin levels (16,6% and 77,2%, resp.). However, baseline prolactin levels measured immediately before the next dialysis session did not change markedly. After cabergoline treatment was started, prolactin levels normalized and galactorrhea disappeared. Thus, dopaminergic inhibition of prolactin secretion might be reduced in patients with end-stage renal disease. This dopaminergic resistance could be an important mechanism of hyperprolactinemia in hemodialysis patients and its subsequent treatment strategies.

## 1. Background

Hyperprolactinemia is the most common cause of galactorrhea in women. In patients with end-stage renal disease (ESRD), the prevalence of hyperprolactinemia in men and women ranges from 30 to 65%. Nevertheless, little is known about the mechanism of hyperprolactinemia in this patient population.

Prolactin has different isoforms with a molecular size ranging from 23 to 150 kDa. The monomeric 23 kDa prolactin isoform is biologically the most active form. One could speculate that large prolactin isoforms accumulate in patients with a reduced renal clearance. However, hyperprolactinemia in patients undergoing renal replacement therapy (RRT) does not consist abundantly of macroprolactin (>50 kDa) isoforms [1]. However, the long-term effect of RRT on prolactin levels has never been studied.

As proof of concept, we evaluated prolactin levels in a female hemodialysis patient with galactorrhea due to hyperprolactinemia who underwent different dialysis modalities to assess the effect of conventional high-flux hemodialysis (HD) and postdilution hemodiafiltration (HDF) on prolactin levels.

## 2. Case Report

A 52-year-old female hemodialysis patient with galactorrhea due to hyperprolactinemia was treated with cabergoline (dopamine D_2_ receptor agonist). The patient had no residual renal function. Because of recent cardiothoracic surgery, cabergoline was discontinued. One month after surgery, prolactin level was only slightly elevated (48.1 *μ*g/l, reference level: 2.2–30.3 *μ*g/l) without any complains of galactorrhea. Cabergoline treatment was not restarted. A few months later, her dialysis modality was switched from HD to HDF because of persistent uremic complaints. Unfortunately, she did not notice any effect of this change after three months and dialysis modality was reversed to HD. Meanwhile sertraline was started because of a depression.

One month after the cessation of HDF, she presented again with complaints of galactorrhea. The patients' thyroid function was normal (TSH: 0.73 mU/l, reference level: 0.36–3.74 mU/l) and she is postmenopausal (FSH: 28.5 U/l, postmenopausal reference level: 19.3–101 U/l; LH: 27.4 U/l, postmenopausal reference level: 8.6–61.8 U/l; estradiol: 128 ng/l; postmenopausal reference level: < 138 ng/l). Prolactin levels immediately before and after a HD session were 131.1 *μ*g/l and 109.3 *μ*g/l, respectively. At the moment of analysis, dialysis vintage was 94 months and dialysis adequacy targets were reached (eKT/V 1.64 and URR 78%). Breast ultrasonography showed no abnormalities and there was no pituitary mass on nuclear magnetic resonance imaging. Diagnosis of hyperprolactinemia due to ESRD in combination with sertraline therapy was made.

**Figure 1:**
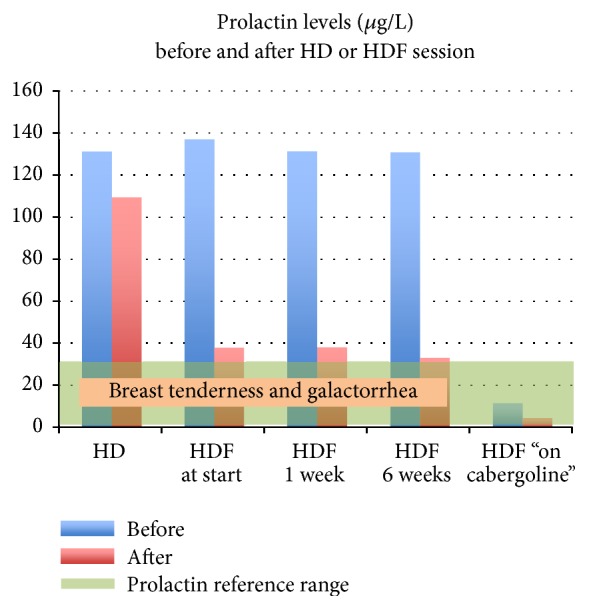
Prolactin levels (*y*-axis) before and after four-hour HD and HDF session (with or without cabergoline). Prolactin reference range: 2.2–30.3 *μ*g/l.

Since galactorrhea reappeared shortly after HDF was switched to HD, we hypothesized that HDF removes prolactin more effectively than HD. Prolactin levels immediately before and after a HDF session were 136.9 *μ*g/l and 38.0 *μ*g/l, respectively. However, after six-week treatment with HDF, galactorrhea persisted and prolactin levels immediately before and after a HDF session were 130.7 *μ*g/l and 33.2 *μ*g/l, respectively. Dialysis adequacy targets on HDF were also reached (eKT/V 1.81 and URR 83%). Treatment with cabergoline was restarted, after which galactorrhea resolved and prolactin levels normalized to 11.5 *μ*g/l and 4.4 *μ*g/l before and after a HDF session, respectively (Figure 1). In all the measured prolactin levels, there was no macroprolactin present.

## 3. Discussion

The increase in prolactin levels in patients on RRT could be partly explained by reduced renal clearance of prolactin [2, 3]. HD is known to remove small molecules (0–0.5 kDa) through diffusion. HDF combines diffusion and convection and improves the clearance of middle-sized molecules (0,5–15 kDa) such as *β*2-microglobulin (11.8 kDa) [4].

Hitherto, no comparison was made between HD and HDF regarding prolactin clearance. In our patient, we found significantly lower prolactin levels immediately after a HDF session compared to a HD session, while dialysis adequacy between both is quasi-similar. However, baseline prolactin levels, measured immediately before a HD or HDF session, did not change markedly and galactorrhea persisted. Stimulation of the D_2_ receptor was needed to obtain physiological prolactin levels and total clinical recovery. Delayed disappearance of galactorrhea after normalization of prolactin could be expected. Al-Husaynei et al. described that galactorrhea disappeared in 100% of all women with hyperprolactinemic amenorrhea after eight-week cabergoline treatment [5].

**Figure 2:**
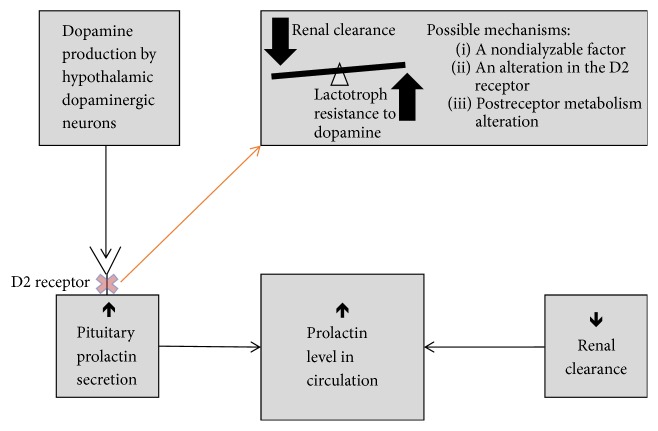
Concept to explain hyperprolactinemia in end-stage renal failure.

An additional factor contributing to sustained hyperprolactinemia in patients with ESRD could be due to an enhanced prolactin secretion by the pituitary. The secretion of prolactin is predominantly regulated by dopaminergic inhibition and this mechanism could be blunted in patients on RRT, also called lactotrophic resistance [2–4]. The mechanism behind that has not been fully elucidated yet (Figure 2). Sievertsen et al. [6] suggested some mechanisms. First, there could be a uremic factor which could not be removed by RRT and interferes with the binding of local dopamine to its receptor. Second, the quality and/or quantity of the dopamine receptors could be changed. Finally, modification in postreceptor metabolism of the dopamine receptor could be considered [6]. In addition, an increase in prolactin level is a known secondary effect of sertraline treatment. However, whether selective serotonin-reuptake inhibitors (SSRIs) and thus sertraline could cause a clinically overt hyperprolactinemia is still controversial [7, 8]. Against that background, Kim and Park describe prevalence of SSRI-induced hyperprolactinemia of 10.9% [7]. A possible mechanism is that a SSRI could act as a dopamine-reuptake inhibitor [7, 8].

## 4. Conclusion

In addition to the reduced renal clearance of prolactin, lactotrophic resistance is a major cause of hyperprolactinemia in patients on RRT. Stimulation of the D_2_ receptor, rather than increased renal clearance alone, is needed for biochemical and clinical response. It merits further investigation to elucidate the underlying mechanism.
